# Factors associated with modern contraceptive use: a comparative analysis between younger and older women in Umlazi Township, KwaZulu-Natal, South Africa

**DOI:** 10.1177/17455065211060641

**Published:** 2021-11-19

**Authors:** Mbuzeleni Hlongwa, Chester Kalinda, Karl Peltzer, Khumbulani Hlongwana

**Affiliations:** 1 Discipline of Public Health, School of Nursing and Public Health, University of KwaZulu-Natal, Durban, South Africa; 2 Burden of Disease Research Unit, South African Medical Research Council, Cape Town, South Africa; 3 University of Global Health Equity, Bill and Joyce Cummings Institute of Global, Kigali, Rwanda; 4 Department of Research Administration and Development, University of Limpopo, Polokwane, South Africa; 5 Department of Psychology, College of Medical and Health Sciences, Asia University, Taichung, Taiwan

**Keywords:** contraceptive use, KwaZulu-Natal, South Africa, termination of pregnancy, unplanned pregnancy, women of reproductive age

## Abstract

**Introduction:**

Unplanned pregnancy continues to be a global reproductive and public health concern among women. This study aimed to investigate whether factors associated with modern contraceptive use differ by age-group among young and older women of reproductive age.

**Methods:**

This was a cross-sectional study conducted among 433 women of reproductive age, with the median age of 25 years (interquartile range: 21–28), and aged between 18 and 49. Data were collected from 10 public health care clinics in Umlazi Township, KwaZulu-Natal, using a structured questionnaire. Data were coded, entered into Epi Data Manager and exported to Stata for analysis. A Pearson’s chi-square test and logistic regression models were employed to assess the level of the association between the predictor and outcome variables, and the p-value of 0.05 or lower was considered statistically significant.

**Results:**

Most women in the sample (n = 351, 81%) had obtained a secondary level of education, while 53% (n = 230) were unemployed and 89% (n = 387) were single. We found that women with secondary level of education (AOR: 2.89, 95% CI: 0.99–5.38) or a tertiary level of education (AOR 3.80, 95% CI: 1.07–3.53) were more likely to use contraceptive methods compared to women with lower education. Women who experienced unplanned pregnancy (AOR 0.51, 95% CI: 0.22–3.79) were more likely to use contraceptives. Women aged 25–49 years who experienced pregnancy, whether planned (AOR 3.87, 95% CI: 1.08–3.89) or unplanned (AOR 3.60, 95% CI: 2.15–4.19), were more likely to use a contraceptive method. Results showed that the level of education (p = 0.942) and whether one experienced unplanned pregnancy (p = 0.913) were not significant predictors of contraceptive use among women aged 18–24 years.

**Conclusion:**

Concerted educational efforts to addressing existing barriers deterring women from accessing contraception among young women are necessary. Different groups of women should be targeted with family planning interventions specific to their needs.

## Introduction

Unplanned pregnancy continues to be a global reproductive and public health concern among women, despite the policies and strategies implemented to improving contraceptive use, globally, as well as in sub-Saharan Africa (SSA).^1^ In South Africa, the percentage of sexually active women using modern contraceptive methods has slightly dropped, from 64% in 2003 to 60% in 2016.^2,3^ Despite the widely available contraceptive methods in public health facilities, the prevalence of contraceptive use by young women remains low,^4^ leading to increased numbers of unplanned pregnancies, sometimes resulting to termination of pregnancies in South Africa.^5^ This may, in part, suggest that accessing family planning services remains a challenge or that negative attitudes towards contraceptive use persist.^6^ The high incidence of unplanned pregnancy is an indication of the unmet needs for contraceptive use in South Africa.^7^ This is worsened by the unprotected sexual activity and early sexual debut among adolescents (15 years and below), who may either be unaware of the available family planning services or simply lack courage to access them for fear of possible moral judgements or even compromised confidentiality in health care clinics.^6,8,9^

Plans and policies aimed at improving contraceptive use have been introduced by the South African government,^10,11^ with emphasis on improving contraceptive use and access to long-acting reversible contraception (LARC) methods receiving support from different policy makers and advocates.^12^ The use of contraceptive methods, such as injectable progestins depot-medroxyprogesterone acetate (DMPA) and norethisterone enanthate (NET-EN), has continued to rise in sub-Saharan Africa region as well as in South Africa.^13^ The injectable contraceptives are largely popular among women and healthcare providers as they are considered to be the most convenient methods in South Africa.^14,15^ However, discontinuation has also been reported with this method due to side effects and costs.^16,17^ About 31% and 28% discontinuation rates have been reported for 3-month injectables and 2-month injectables, respectively.^18^ In South Africa, among all women who use contraception use a modern method, accounting for 24% injectables, 9% male condom and 8% each for contraceptive pills and female sterilization among women in union.^18^

Modern contraceptive use among young women is inconsistent, irrespective of their marital status.^19^ When the use of contraceptives is inconsistent, for one reason or the other, the likelihood of unplanned pregnancies would also increase. Much of the research literature about contraceptive use has been generalized among all women, with limited comparative analysis to consider the contribution of age in the use of contraceptives. Therefore, understanding the key factors associated with contraceptive use among women of different age-groups is important to the development of effective age-sensitive family planning programmes and devising effective policies in South Africa. As such, this study aimed to investigate factors associated with contraceptive use among women of reproductive age in Umlazi Township, KZN province, South Africa. Data were further stratified by age categories, 18–24 years and 25–49 years.

## Methods

### Study setting

Umlazi Township, which is located in the province of KZN, is the second largest populated township in South Africa, with an estimated population of more than half a million people.^20^ The township is part of the EThekwini Metro, which has the largest number of people on lifelong anteretroviral therapy (ART) in the province.^21^ Umlazi has 10 public health care clinics and one public health hospital. All 10 public health care clinics in Umlazi participated in the study. On average, these clinics combined serve more than 50,000 clients per month.

### Study design, participants and sampling

An analytical cross-sectional study was conducted between November 2018 and April 2019. All potentially eligible women who accessed general health care services at the time of data collection were approached, introduced to the study and invited to participate. A total of 433 women aged between 18 and 49 years, who were sexually active, residents of Umlazi Township and accessing health care services at any of the 10 Umlazi Township clinics, were recruited for the study. A sample size of 300 was required to estimate the proportion of women using contraception in Umlazi Township to within 8% with a probability of 95% and assuming 50% prevalence. To adjust for the clustering effect of clinic, a design effect of 1.25 was included. The minimum final sample required for this study was therefore 375 for women respondents. The sample proportional to size (SPS) was used to ensure that sample sizes varied and was reflective of patient volume per each health facility. Due to time limitation, a convenience sampling technique was used to enroll women attending clinic on each day of the week during data collection period. The study participants were only approached for participation after services had been rendered by the clinic.

### Study instrument and data collection

A structured questionnaire was designed in English and translated into IsiZulu (local) language. Two experienced research assistants (RAs) were recruited and trained on the consent process and questionnaire administration. These two RAs were competent in both English and IsiZulu languages. The training and language competency enabled them to properly introduce the study to individual potential participants and secure the consent prior to administering the questionnaires. The instrument was pre-tested on 10 participants who did not form part of the study, prior to data collection. The study instrument (a structured questionnaire) was administered by research assistants during clinic operating times. The questionnaire asked for information containing the demographic and socioeconomic characteristics of the study participants, awareness of contraceptive methods, the use of contraceptives and information related to sexual behaviour. A series of quality assurance processes were implemented to ensure that data quality was not compromised but preserved, including data validation, data cleaning, questionnaire verification, as well as ensuring that questionnaires were tested for consistency. Administered questionnaires were checked by the principal investigator to ensure quality assurance of collected data and completeness of questionnaires. The inclusion criteria were (a) women of reproductive age (18–49 years) who visited the health care facilities for any services at any day of the week during data collection, and (b) women residing in Umlazi Township. Women who were either under the age of 18 years, pregnant, or sexually inactive were excluded from the study. We also excluded women who were not able or willing to sign informed consent.

### Ethics

Ethical approval to conduct this study was obtained from the Biomedical Research Ethical Committee (BREC) at the University of KwaZulu-Natal (Ref No: BE424/18). The National Health Research Database (NHRD) from the KZN Provincial Department of Health (Ref No: KZ_2018009_013), and The EThekwini District’s Ethical Review Committee also approved the study. Gatekeeper permissions were obtained from the participating individual facilities prior to data collection. To ensure the privacy and confidentiality of respondents, no personal identifiers were captured in the questionnaires. Likewise, a written informed consent was obtained from the study participants prior to their enrolment. The research assistants went through the informed consent with the potential participants in a language preferred by the participants. The study adhered to sound ethical standards including confidentiality, voluntariness of participation, option to terminate participation and full disclosure of the research process.

### Data analysis

Data were coded, entered into Epi Data Manager (version 4.6)^22^ and exported to Stata version 15^23^ for cleaning and analysis. The dependent dichotomous variable was the use of any contraceptive method currently. The use of contraceptive methods as well as other socio-demographic characteristics was summarized using descriptive statistics. In estimating the influence of socio-demographic characteristics on the use of contraceptives, we stratified participants by age categories, 18–24 years and 25–49 years. A Pearson’s chi-square test was used to determine the association between socio-demographic characteristics and the use of contraceptives. Statistically significant variables in the bivariate analysis were used as predictors in the multivariate logistic regression. The results were expressed as odds ratio (OR) with their 95% confidence interval (CI) and statistical significance level of p < 0.05.

## Results

### Background characteristics of study participants

The study included 433 female participants (Table 1). The median age of respondents was 25 years (interquartile range (IQR): 21–28)), and their ages ranged between 18 and 49 years. Most women in the sample (n = 351, 81%) had obtained a secondary level of education, accounting for 80% (n = 181) among women aged 18–24 years and 83% (n = 170) among 25–49 years age-group. Among women aged 18–25 years, 44% (n = 100) were unemployed and 46% (n = 105) were studying; 63% (n = 130) of women aged 25–49 years were unemployed. Most women in the sample (n = 387, 89%) were single, accounting for 94% (n = 214) among women aged 18–24 years and 84% (n = 173) among women aged 25–49 years. Almost all women in the sample were Black African (n = 427, 99%).

**Table 1.** Background characteristics of study participants by age group, Umlazi Township, KwaZulu-Natal, 2019.

Characteristics	Age group		Total (18–49 years) n = 433
	18–24 years n = 227	25–49 years n = 206	
Age (years)			
Median (IQR)	21 (19–23)	31 (26–33)	25 (21–28)
Level of education, n (%)			
Primary	1 (0,4)	8 (4)	9 (2)
Secondary	181 (80)	170 (83)	351 (81)
Tertiary	44 (19)	27 (13)	71 (16)
Employment status, n (%)			
Unemployed	100 (44)	130 (63)	230 (53)
Employed	22 (10)	60 (29)	82 (19)
Studying	105 (46)	12 (6)	117 (27)
Marital status, n (%)			
Married/living with partner	7 (3)	30 (15)	37 (9)
Single	214 (94)	173 (84)	387 (89)
Separated	1 (0,4)	2 (1)	3 (1)
Population group, n (%)			
Black African	225 (99)	202 (98)	427 (99)
Coloured/Asian	1 (0,4)	2 (1)	3 (1)

IQR: interquartile range.

a The total does not add up to 433 because of missing data caused by non-reporting from participants. Variable categories present column percentages.

### Knowledge and current use of contraception by method type

Among all women in the sample, 87% of those aged 18–24 years and 93% of those aged 24–49 years had some form of knowledge about injectable contraceptive method (Table 2). About 52% and 49% of women aged 18–24 years and 25–49 years were reportedly using injectable contraceptives, respectively. Approximately 74% and 76% of women aged 18–24 years and 25–49 years, respectively, had some knowledge about condoms, while 52% and 49% of women aged 18–24 years and 25–49 years, respectively, were using condoms as contraception at the time of data collection. Half (50%) of women aged 18–24 years and 56% of women aged 25–49 years had some knowledge about a pill contraceptive method, while 3% and 4% were using it at the time of the study, respectively.

**Table 2.** Distribution of knowledge and use of contraception by method type, Umlazi Township, KwaZulu-Natal, 2019.

Method type	Knowledge		Use during data collection	
	18–24 years n = 227	25–49 years n = 206	18–24 years n = 191	25–49 years n = 173
Abstinence	5 (2)	6 (3)	8 (4)	9 (5)
Condom	167 (74)	157 (76)	100 (52)	85 (49)
Diaphragm/foam/jelly	2 (1)	0 (0)	1 (1)	0 (0)
Female sterilization	4 (2)	8 (4)	2 (1)	10 (6)
Implant	51 (22)	38 (18)	7 (4)	0 (0)
Injection	197 (87)	192 (93)	104 (54)	109 (63)
IUD	37 (16)	46 (22)	2 (1)	6 (3)
Male sterilization	0 (0)	0 (0)	0 (0)	1 (1)
Pill	113 (50)	115 (56)	5 (3)	7 (4)
Withdrawal	3 (1)	5 (2)	0 (0)	4 (2)

a Method type present column percentages.

Of the 433 respondents who participated in the study, 84% (n = 364) were using a contraceptive method (Table 3). Among women who had obtained secondary level of education, contraceptive users accounted for 44% (n = 153) and 41% (n = 144) among those aged 18–24 years and 25–49 years, respectively. Contraceptive users accounted for 47% (n = 180) and 37% (n = 144) among single women aged 18–24 years and 25–49 years, respectively. Approximately 14% (n = 16) of women aged 18–24 years who were studying were not using a contraceptive method. Among women aged 25–49 years who were unemployed, 46% (n = 106) were using a contraceptive method. Table 3 shows more findings that are detailed.

**Table 3.** Characteristics of participants stratified by age group and contraceptive use status, Umlazi Township, KwaZulu-Natal, South Africa, 2019.

Characteristics	Contraceptive users		Non-contraceptive users		Total (18–49 years) n = 433
	18–24 years n = 191	25–49 years n = 173	18–24 years n = 36	25–49 years n = 33	
Level of education, n (%)					
Primary	0 (0)	5 (56)	1 (11)	3 (33)	9 (2)
Secondary	153 (44)	144 (41)	27 (8)	26 (7)	350 (81)
Tertiary	37 (52)	24 (34)	7 (10)	3 (4)	71 (16)
Marital status, n (%)					
Married	5 (14)	27 (73)	2 (5)	3 (8)	37 (9)
Single	180 (47)	144 (37)	33 (9)	29 (8)	386 (89)
Separated	1 (33)	1 (33)	0 (0)	1 (33)	3 (1)
Employment status, n (%)					
Unemployed	84 (37)	106 (46)	16 (7)	24 (10)	230 (53)
Employed	19 (23)	53 (65)	3 (4)	7 (9)	82 (19)
Studying	88 (76)	11 (9)	16 (14)	1 (1)	116 (27)
Ever diagnosed with HIV, n (%)					
No	154 (53)	91 (31)	26 (9)	18 (6)	289 (67)
Yes	28 (24)	73 (62)	4 (3)	12 (10)	117 (27)
HIV status of sexual partner, n (%)					
Do not know	26 (39)	34 (52)	2 (3)	4 (6)	66 (15)
Negative	137 (54)	83 (33)	25 (10)	10 (4)	255 (59)
Positive	17 (25)	40 (59)	3 (4)	8 (12)	68 (16)
Experienced hitting/slapping by partner, n (%)					
Agree	25 (56)	14 (31)	3 (7)	3 (7)	45 (10)
Neutral	4 (44)	4 (44)	1 (11)	0 (0)	9 (2)
Disagree	142 (45)	130 (41)	24 (8)	18 (6)	314 (73)
Ever been pregnant before, n (%)					
No	55 (66)	12 (14)	8 (10)	8 (10)	83 (19)
Yes	135 (39)	157 (46)	26 (8)	25 (7)	343 (79)
Terminated pregnancy, n (%)					
No	167 (43)	157 (41)	31 (8)	29 (8)	384 (89)
Yes	10 (53)	7 (37)	2 (11)	0 (0)	19 (4)
Partner has control over sex, n (%)					
Agree	40 (51)	31 (40)	5 (6)	2 (3)	78 (18)
Neutral	72 (43)	76 (45)	11 (7)	10 (6)	169 (39)
Disagree	61 (50)	40 (33)	12 (10)	8 (7)	121 (28)
Partner has control over condom use, n (%)					
Agree	31 (51)	22 (36)	5 (8)	3 (5)	61 (14)
Neutral	73 (44)	75 (45)	8 (5)	10 (6)	166 (38)
Disagree	68 (48)	52 (36)	15 (10)	8 (6)	143 (33)
Number of male sexual partners (past 3 months), n (%)					
One	167 (48)	139 (40)	27 (8)	14 (4)	347 (80)
More than one	11 (37)	14 (47)	3 (10)	2 (7)	30 (7)
Age at first sex, n (%)					
12–17 years	89 (55)	47 (29)	15 (9)	12 (7)	163 (38)
18–24 years	92 (39)	111 (47)	18 (8)	17 (7)	238 (55)

HIV: human immunodeficiency virus.

a Variable categories present row percentages.

### Factors associated with contraceptive use in univariate and multivariate analyses

Contraceptive use among women in Umlazi Township, KwaZulu-Natal, showed significant association with two factors at univariate analysis (Table 4), and these were as follows: having secondary level of education (OR 2.18, 95% CI: 1.17–4.24) or tertiary level of education (OR 2.11, 95% CI: 1.12–2.33), and women who had an unplanned pregnancy (OR 0.39, 95% CI: 0.21–0.74). These variables were included in the multivariate analysis and disaggregated by age group.

**Table 4.** Factors associated with contraceptive use in univariate and multivariate analysis, Umlazi Township, KwaZulu-Natal, South Africa, 2019.

Determinants	Odds ratios (unadjusted)	P-value	95% conf. interval	Odds ratios (adjusted)	P-value	95% CI
Education level						
Primary (ref)						
Secondary	2.18	**0.029**	1.17—4.24	2.89	**0.053**	0.99—5.38
Tertiary	2.11	**0.035**	1.12 2.33	3.80	**0.040**	1.07—3.53
Employment status						
Unemployed (ref)						
Employed	1.52	0.273	0.72—3.19			
Studying	1.23	0.518	0.66—2.27			
Marital status						
Married (ref)						
Single	0.82	0.685	0.31—2.17			
Separated	0.31	0.377	0.02—4.12			
Ever been pregnant						
No (ref)						
Yes	1.37	0.324	0.73—2.54			
Planned pregnancy						
Not applicable (ref)						
Yes	0.60	0.163	0.29—3.82	0.63	0.214	0.30—2.31
No	0.39	**0.004**	0.21—0.74	0.51	**0.008**	0.22—3.79
Ever terminated pregnancy						
Yes (ref)						
No	1.57	0.551	0.35—6.99			
Ever diagnosed with HIV						
No (ref)						
Yes	1.13	0.690	0.61—2.10			
HIV status of sexual partner						
Do not know (ref)						
Negative	0.63	0.318	0.25—1.56			
Positive	0.52	0.224	0.18—1.49			
Experienced hitting/slapping by partner						
Neutral (ref)						
Agree	0.81	0.856	0.08—6.11			
Disagree	0.80	0.844	0.09—6.64			
Partner has control over sex						
Neutral (ref)						
Agree	1.44	0.428	0.58—3.54			
Disagree	0.72	0.324	0.37—1.39			

HIV: human immunodeficiency virus; Ref: reference category.

In a multivariate analysis (Table 4), both variables were significant predictors of contraceptive use among women in Umlazi Township, KwaZulu-Natal. The odds of contraceptive use (AOR: 2.89, 95% CI: 0.99–5.38) among women who had attained secondary level of education was 2.89 times higher compared to those with primary level of education. For women who had attained tertiary level of education compared to those who had primary level of education, the odds of contraceptive use were 3.80 times higher (AOR 3.80, 95% CI: 1.07–3.53). The odds of using contraceptives were significantly higher among women who had experienced unplanned pregnancy (AOR 0.51, 95% CI: 0.22–3.79).

Further analysis for variables that had been found to be significant predictors in a multivariate analysis were disaggregated by age-group (Table 5). The analysis indicated that the level of education (*p* = 0.942) and whether one experienced unplanned pregnancy (*p* = 0.913), were not significant predictors of contraceptive use among women aged 18–24 years. Among women aged 25–49 years, level of education was not significantly associated with contraceptive use (*p* = 0.307). Women aged 25–49 years who had experienced pregnancy, whether planned (AOR 3.87, 95% CI: 1.08–3.89) or unplanned (AOR 3.60, 95% CI: 2.15–4.19) were significantly more likely to use a contraceptive method.

**Table 5.** Multivariate analysis disaggregated by age group, Umlazi Township, KwaZulu-Natal, South Africa, 2019.

Determinants	18–24 years			25–49 years		
	OR	P-value	95% conf. interval	OR	P-value	95% conf. interval
Education level						
Primary (ref)						
Secondary	1.03	0.942	0.41 2.59	2.31	0.307	0.46 3.55
Tertiary				5.05	0.111	0.69 3.92
Planned pregnancy						
Not applicable (ref)						
Yes	1.07	0.913	0.30 3.89	3.87	**0.038**	1.08 3.89
No	1.51	0.336	0.65 3.49	3.60	**0.001**	2.15 4.19

OR: odds ratios; Ref: reference category.

## Discussion

This study analysed the factors associated with contraceptive use among younger (aged 18–24 years) and older (aged 25–49 years) women in Umlazi Township, KZN province, South Africa. Eighty-four per cent (84%) of participants were currently using a contraceptive method. We found that women who had obtained a secondary level of education or a tertiary level of education were significantly more likely to use a contraceptive method compared to women who had obtained a primary level of education. Women who had experienced unplanned pregnancy were more likely to use a contraceptive method. After disaggregating by age group, results showed that the level of education (p = 0.942) and having experienced unplanned pregnancy (p = 0.913), were not significant predictors of contraceptive use among women aged 18–24 years. Women aged 25–49 years who had experienced pregnancy, whether planned or unplanned were significantly more likely to use a contraceptive method.

The World Health Organization reported that contraceptive use among adolescents is low, given the estimated 220 000 unplanned pregnancies among HIV positive women in South Africa.^24^ Similar to our findings, a study conducted in South Africa reported that being HIV positive, having been diagnosed with STI in the past 12 months and early sexual debut were not significant predictors of contraceptive use among younger women.^25^ This poor contraceptive use may be explained by the fact that young women face many barriers such as lack of information, costs, unavailability of methods of choice, side effects, which hinder their access and utilization of family planning services in public health care facilities.^6,26–28^ Younger women are also discouraged to seek contraceptive methods due to fear of being judged, stigma and negative attitudes from health care providers.^15^

The results of this study support what has been found in other settings, with older women having higher odds of using contraceptives compared to young women.^7^ Among women aged 25–49 years, the association between contraceptive use and women who experienced unplanned pregnancies may be attributed to the fact that these women may be aiming to avoid occurrence of similar mistake. However, our study also found that women aged 25–49 years who had planned pregnancies were also significantly associated with contraceptive use. Contraceptive use has also been linked with having previously been pregnant among South African women.^4^ This finding may suggest that older women may already have obtained the desired number of children or desire child-spacing, hence the need for using contraceptive methods to prevent another pregnancy.

Education was a significant predictor of contraceptive use in both groups of women at univariate and multivariate analysis, although the reverse was observed after stratifying data by age. It has been shown in another study that women with high level of education are more likely to use contraceptives compared to women with low level of education,^6^ as education is often associated with freedom to make independent decisions.^29^ High level of education is a strong determinant for contraceptive use, because education may expose women to information useful for appropriate decision-making regarding their reproductive health needs.^6^ An increasing level of education has also been linked with improved use of contraception among sexually active women, from 44% among women with no formal education to more than 60% among women who had attained a secondary level of education.^18^ These findings suggest that empowering women through education is important for improving contraceptive use.

While this study is making an important contribution in the field of reproductive health, the following limitations should be noted. Given that the sampling frame for this study was limited to women seeking healthcare services in public health care clinics in Umlazi Township, women who do not use public health care services or use them less frequently were excluded and/or under-represented in the sample. Our findings showed a high proportion of women (84%) using contraception. While data was collected from Monday to Friday, our study may have over sampled women attending maternal and child health services. This study sought self-reported sexual health information from participants, thereby making the findings vulnerable to social desirability bias. Furthermore, information deemed to have potential for leading to value judgements may have been withheld by the participants. Older participants may have been unable to recall the age at which they had sexual debut.

## Conclusion

This study showed that the factors associated with contraceptive use differ between young and older women. Concerted educational efforts to addressing existing socioeconomic and structural barriers related to contraceptive use among young women in Umlazi Township are necessary. Such efforts may be accompanied by educational programmes aimed at improving contraceptive use. Results further suggest that family planning interventions should be tailored to address the specific needs of different age groups of women, as their needs may vary.

## Supplemental Material

sj-docx-1-whe-10.1177_17455065211060641 – Supplemental material for Factors associated with modern contraceptive use: a comparative analysis between younger and older women in Umlazi Township, KwaZulu-Natal, South Africa Click here for additional data file. Supplemental material, sj-docx-1-whe-10.1177_17455065211060641 for Factors associated with modern contraceptive use: a comparative analysis between younger and older women in Umlazi Township, KwaZulu-Natal, South Africa by Mbuzeleni Hlongwa, Chester Kalinda, Karl Peltzer and Khumbulani Hlongwana in Women’s Health
