# Supplementary material for: Factors associated with modern contraceptive use: a comparative analysis between younger and older women in Umlazi Township, KwaZulu-Natal, South Africa
Source: Womens Health (Lond). 2021 Nov 19;17:17455065211060641. doi: 10.1177/17455065211060641 (PMC8606954; doi:10.1177/17455065211060641)
Supplement: sj-docx-1-whe-10.1177_17455065211060641 – Supplemental material for Factors associated with modern contraceptive use: a comparative analysis between younger and older women in Umlazi Township, KwaZulu-Natal, South Africa [file sj-docx-1-whe-10.1177_17455065211060641.docx]

**Title: FACTORS INFLUENCING CONTRACEPTIVE USE AND SEXUAL BEHAVIOUR AMONG WOMEN OF REPRODUCTIVE AGE IN UMLAZI TOWNSHIP, KWAZULU-NATAL PROVINCE, SOUTH AFRICA.**

**SECTION 1: DEMOGRAPHIC CHARACTERISTICS**

|  | **1.1 What was your age (in years) in your last birthday?** | | | |
| --- | --- | --- | --- | --- |
| 1. |  | Years |  |  |
|  | **1.2 What is your gender?** | | |  |
| 1. | Born Female | | | 1 |
| 2. | Born Male | | | 2 |
| 3. | Other (please specify) | | | 3 |
|  | **1.3 What is your home language?** | | | |
| 1. | IsiZulu | | | 1 |
| 2. | English | | | 2 |
| 3. | Afrikaans | | | 3 |
| 4. | Ndebele | | | 4 |
| 5. | Venda | | | 5 |
| 6. | Tsonga | | | 6 |
| 7. | Sepedi | | | 7 |
| 8. | Swati | | | 8 |
| 9. | Xhosa | | | 9 |
| 10. | Sotho | | | 10 |
| 11. | Tswana | | | 11 |
| 12. | Other (Please specify): | | | 12 |
|  | **1.4 Which race group do you consider yourself to belong to?** | | | |
| 1. | Black/African | | | 1 |
| 2. | Coloured | | | 2 |
| 3. | White | | | 3 |
| 4. | Asian/Indian | | | 4 |
| 5. | Other (Please specify) | | | 5 |
|  | **1.5 What is the highest level of education you have passed?** | | | |
| 1. | No formal education | | | 1 |
| 2. | Grade 1 | | | 2 |
| 3. | Grade 2 | | | 3 |
| 4. | Grade 3 | | | 4 |
| 5. | Grade 4 | | | 5 |
| 6. | Grade 5 | | | 6 |
| 7. | Grade 6 | | | 7 |
| 8. | Grade 7 | | | 8 |
| 9. | Grade 8 | | | 9 |
| 10. | Grade 9 | | | 10 |
| 11. | Grade 10 | | | 11 |
| 12. | Grade 11 | | | 12 |
| 13. | Grade 12 | | | 13 |
| 14. | Diploma/ Degree/ other post school – incomplete | | | 14 |
| 15. | Diploma/ Degree/ other post school – complete | | | 15 |
| 16. | Diploma/ Degree graduate | | | 16 |
| 17 | Postgraduate Diploma/ Degree | | | 17 |
|  | **1.6 What is your current marital status?** | | |  |
| 1. | Legally married | | | 1 |
| 2. | Traditionally married | | | 2 |
| 3. | Living with man in union | | | 3 |
| 4. | Never married/Single | | | 4 |
| 5. | Divorced | | | 5 |
| 6. | Married but separated | | | 6 |
| 7. | Widowed | | | 7 |

**SECTION 2: ECONOMIC FACTORS**

|  | **2.1 Have you done any paid work in the last 12 months?** | |
| --- | --- | --- |
| 1. | No | 0 |
| 2. | Yes | 1 |

|  | **2.2 Which of the following describes your current employment status?** | | | |  |
| --- | --- | --- | --- | --- | --- |
| 1. | Unemployed | 1 |  |  |  |
| 2. | Employed part-time | 2 |  |  |  |
| 3. | Employed full-time | 3 |  |  |  |
| 4. | Self-employed | 4 |  |  |  |
| 5. | Studying | 5 |  |  |  |
|  | **2.3 Please indicate which of the following are your source(s) of income. (Please answer this question whether or not you are working).** | | | | |
|  |  | | | Yes | No |
| 1. | Work/self-employment | | | 1 | 0 |
| 2. | Spouse/partner | | | 1 | 0 |
| 3. | Parents | | | 1 | 0 |
| 4. | Brothers and/or sisters | | | 1 | 0 |
| 5. | Children | | | 1 | 0 |
| 6. | Child Support Grant | | | 1 | 0 |
| 7. | State Old Age Pensions | | | 1 | 0 |
| 8. | Disability Grant | | | 1 | 0 |
| 9. | Care Dependency Grant | | | 1 | 0 |
| 10. | Foster Care Grant | | | 1 | 0 |
| 11. | Grants-in-Aid | | | 1 | 0 |
| 12. | Workman’s Compensation Fund | | | 1 | 0 |
| 13. | Other (Please specify) | | | 1 | 0 |

**SECTION 3: HEALTH**

|  | **3.1 In general, how would you rate the state of your health?** |  |
| --- | --- | --- |
| 1. | Excellent | 1 |
| 2. | Very Good | 2 |
| 3. | Good | 3 |
| 4. | Fair | 4 |
| 5. | Poor | 5 |

|  | **3.2 Please choose the number that best describes the extent to which each of the following statements is true or false for you.** | | | | | |
| --- | --- | --- | --- | --- | --- | --- |
|  |  | Definitely true | Mostly true | Not sure | Mostly false | Definitely false |
| 1. | I am somewhat ill | 1 | 2 | 3 | 4 | 5 |
| 2. | I am as healthy as anybody I know | 1 | 2 | 3 | 4 | 5 |
| 3. | My health is excellent | 1 | 2 | 3 | 4 | 5 |
| 4. | I have been feeling unhealthy lately | 1 | 2 | 3 | 4 | 5 |

**SECTION 4: USE OF CONTRACEPTIVES**

|  | **4.1 How old were you when you had your first period?** |  |
| --- | --- | --- |
| 1. | Less than ten years old | 1 |
| 2. | Ten to fifteen years old | 2 |
| 3. | Sixteen to twenty years old | 3 |
| 4. | Beyond twenty years old | 4 |

|  | **4.2 Have you ever used anything or tried in any way to delay or avoid getting pregnant?** | |
| --- | --- | --- |
| 1. | No | 0 |
| 2. | Yes | 1 |

**4.3 Which is the contraceptive method are you most familiar with to delay or avoid getting pregnant?**

| 1. | Pill | 1 |
| --- | --- | --- |
| 2. | IUD | 2 |
| 3. | Injections | 3 |
| 4. | Diaphragm/foam/jelly | 4 |
| 5. | Condom | 5 |
| 6. | Female sterilisation | 6 |
| 7. | Male sterilisation | 7 |
| 8. | Calendar/rhythm | 8 |
| 9. | Withdrawal | 9 |
| 10. | Traditional herbs/remedies | 10 |
| 11. | Abstinence | 11 |
| 12. | Other (Please specify) | 12 |
| 13. | None | 98 |

**4.4 Which is the main method that you are using now to delay or avoid getting pregnant?**

| 1. | Pill | 1 |
| --- | --- | --- |
| 2. | IUD | 2 |
| 3. | Injections | 3 |
| 4. | Diaphragm/foam/jelly | 4 |
| 5. | Condom | 5 |
| 6. | Female sterilisation | 6 |
| 7. | Male sterilisation | 7 |
| 8. | Calendar/rhythm | 8 |
| 9. | Withdrawal | 9 |
| 10. | Traditional herbs/remedies | 10 |
| 11. | Abstinence | 11 |
| 12. | Other (Please specify) | 12 |
| 13. | None | 98 |
|  | **4.5 For how long have you used this method?** |  |
| 1. |  | Years |
| 2. |  | Months |
| 3. | 99 | Not applicable |

|  | **4.6 Which are the methods that you have used in the past to delay or avoid getting pregnant?** | Yes | No |
| --- | --- | --- | --- |
| 1. | Pill | 1 | 0 |
| 2. | IUD | 1 | 0 |
| 3. | Injections | 1 | 0 |
| 4. | Diaphragm/foam/jelly | 1 | 0 |
| 5. | Condom | 1 | 0 |
| 6. | Female sterilisation | 1 | 0 |
| 7. | Male sterilisation | 1 | 0 |
| 8. | Calendar/rhythm | 1 | 0 |
| 9. | Withdrawal | 1 | 0 |
| 10. | Traditional herbs/remedies | 1 | 0 |
| 11. | Abstinence | 1 | 0 |
| 12. | Other (Please specify) | 1 | 0 |
| 13. | Unsure | 1 | 0 |
| 14. | None | 1 | 0 |
|  | **4.7 Where do/did you obtain the method you are using currently?** | |  |
| 1. | Government Hospital | 1 |  |
| 2. | Government Clinic | 2 |  |
| 3. | Community Health Centre | 3 |  |
| 4. | Family Planning Clinic | 4 |  |
| 5. | Private Hospital | 5 |  |
| 6. | Private Clinic | 6 |  |
| 7. | Private Doctor | 7 |  |
| 8. | Mobile clinic | 8 |  |
| 9. | Pharmacy/Chemist | 9 |  |
| 10. | Traditional healer | 10 |  |
| 11. | Faith healer | 11 |  |
| 12. | Don’t know | 12 |  |
| 13. | Other (Please specify) | 13 |  |
| 14. | Not applicable | 99 |  |

|  | **4.8 From whom did you first get information about methods to avoid or delay pregnancy? (Circle as many as apply)** | | |
| --- | --- | --- | --- |
| 1. | Mother | 1 | 0 |
| 2. | Sister | 1 | 0 |
| 3. | Father | 1 | 0 |
| 4. | Other Relative | 1 | 0 |
| 5. | Friend | 1 | 0 |
| 6. | Teacher | 1 | 0 |
| 7. | Nurse | 1 | 0 |
| 8. | Doctor | 1 | 0 |
| 9. | Social Worker | 1 | 0 |
| 10. | Poster/Leaflet/Magazine | 1 | 0 |
| 11. | Radio/Television | 1 | 0 |
| 12. | Other (Please specify) | 1 | 0 |
|  | \|  \| **4.9 How old were you when you first used something to avoid or delay getting pregnant?** \| \| \| --- \| --- \| --- \| \| 1. \|  \| Years \| \| 2. \| 99 \| Not applicable \|   **4.9 Have your parent(s) or guardian(s) ever given you advice on contraceptives or explain how to use them?** | |  |
| 1. | No | 0 |  |
| 2. | Yes | 1 |  |

|  | **4.10 Please indicate how strongly you agree or disagree with the following statements.** | Strongly disagree | Disagree | Not sure | Agree | Strongly agree |
| --- | --- | --- | --- | --- | --- | --- |
| 1. | Condoms are easily available | 1 | 2 | 3 | 4 | 5 |
| 2. | The Pill is easily available | 1 | 2 | 3 | 4 | 5 |
| 3. | Injectable contraception is easily available | 1 | 2 | 3 | 4 | 5 |

|  | **4.11 Choose the reason(s) why you are using contraception.** | Yes | No |
| --- | --- | --- | --- |
| 1. | My parents told me to | 1 | 0 |
| 2. | All my friends do it | 1 | 0 |
| 3. | My teacher told me to | 1 | 0 |
| 4. | I saw it on TV | 1 | 0 |
| 5. | Healthcare worker told me to | 1 | 0 |
| 6. | Other (please specify) | 1 | 0 |

|  | **4.12 Choose the reason(s) why you are NOT using contraception.** | Yes | No |
| --- | --- | --- | --- |
| 1. | All my friends are not using it | 1 | 0 |
| 2. | Sex feels better without a condom | 1 | 0 |
| 3. | It is difficult to get hold of contraception | 1 | 0 |
| 4. | Contraception is expensive | 1 | 0 |
| 5. | It is against my religion | 1 | 0 |
| 6. | It is against my culture | 1 | 0 |
| 7. | My boyfriend doesn’t want me to use it | 1 | 0 |
| 8. | It’s a nuisance | 1 | 0 |
| 9. | Contraception has side effects | 1 | 0 |
| 10. | I don’t know what contraception is | 1 | 0 |
| 11. | Other (please specify) | 1 | 0 |

| **SECTION 5: SEXUAL BEHAVIOUR** |  |
| --- | --- |

|  | **5.1 When was the last time you had sex, if ever?** |  |
| --- | --- | --- |
| 1. | Never | 0 |
| 2. | Within the last week | 1 |
| 3. | Within the last month | 2 |
| 4. | More than one month ago | 3 |

**IF YOU HAVE NEVER HAD SEX, PLEASE GO TO SECTION 9**

|  | **5.2 Who did you last have sex with?** |  |  |
| --- | --- | --- | --- |
| 1. | Husband | 1 |  |
| 2. | Boyfriend | 2 |  |
| 3. | Other regular partner | 3 |  |
| 4. | Casual acquaintance | 4 |  |
| 5. | Someone just met | 5 |  |
| 6. | Other (Please specify) | 6 |  |
|  | **5.3 How old were you when you first had sex?** |  | |
| 1. |  | Years | |
| 2. | 99 | Not applicable | |

|  | **5.4 How frequent did you have sexual intercourse in the past month?** |  |
| --- | --- | --- |
| 1. | 0 | 0 |
| 2. | 1-5 times | 1 |
| 3. | 6 times or more | 2 |

|  | **5.5 Have you ever been treated for an STI?** |  |
| --- | --- | --- |
| 1. | No | 0 |
| 2. | Yes | 1 |

|  | **5.6 Have you been diagnosed with an STI in the past 12 months?** |  |
| --- | --- | --- |
| 1. | No | 0 |
| 2. | Yes | 1 |

|  | **5.7 What is the total number of sexual partners you have had in the past three months?** | |
| --- | --- | --- |
| 1. | None 0 | 0 |
| 2. | 1 | 1 |
| 3. | 2-3 | 2 |
| 4. | 4-5 | 3 |
| 5. | 6-7 | 4 |
| 6. | 8-9 | 5 |
| 7. | More than 9 | 6 |

|  | **5.8 How often have you had sex under the influence of alcohol in the past three months?** | |
| --- | --- | --- |
| 1. | Never | 0 |
| 2. | 1-3 times | 1 |
| 3. | 4-6 times | 2 |
| 4. | 7-9 times | 3 |
| 5. | 10-12 times | 4 |
| 6. | More than 12 times | 5 |
| 7. | Not applicable | 9 |

**SECTION 6: USE OF CONDOMS**

|  | **6.1 How often have you used condoms with your spouse or regular partner(s) in the past 3 months?** | |
| --- | --- | --- |
| 1. | Never | 0 |
| 2. | Seldom | 1 |
| 3. | Sometimes | 2 |
| 4. | Always | 3 |
| 5. | Not applicable (respondent had no spouse or regular partner in the past three months) | 9 |

|  | **6.2 How frequently have you used condoms with casual partners in the past 3 months?** | |
| --- | --- | --- |
| 1. | Never | 0 |
| 2. | Seldom | 1 |
| 3. | Sometimes | 2 |
| 4. | Always | 3 |
| 5. | Not applicable (respondent had no spouse or regular partner in the past three months) | 9 |

|  | **6.3 The last time you had sex, was a condom used?** |  |
| --- | --- | --- |
| 1. | No | 0 |
| 2. | Yes | 1 |
| 3. | Don’t know | 2 |
| 4. | Not applicable | 9 |

|  | **6.4 Why did you not use a condom the last time you had sex?** |  |  |  |
| --- | --- | --- | --- | --- |
|  |  | Yes | No | Not Applicable |
| 1. | I did not want to use a condom | 1 | 0 | 9 |
| 2. | I did not need to use a condom | 1 | 0 | 9 |
| 3. | I did not like condoms | 1 | 0 | 9 |
| 4. | I did not know about condoms | 1 | 0 | 9 |
| 5. | I did not have a condom | 1 | 0 | 9 |
| 6. | Other (Please specify) | 1 | 0 | 9 |
| 7. | I used a condom the last time I had sex | 1 | 0 | 9 |

|  | **6.5 Where can you get condoms from?** | Yes | No |
| --- | --- | --- | --- |
| 1. | Government Hospital | 1 | 0 |
| 2. | Day Hospital/Clinic | 1 | 0 |
| 3. | Community Health Centre | 1 | 0 |
| 4. | Family Planning Clinic | 1 | 0 |
| 5. | Mobile Clinic | 1 | 0 |
| 6. | Community Health Worker | 1 | 0 |
| 7. | Private Hospital/Clinic | 1 | 0 |
| 8. | Pharmacy | 1 | 0 |
| 9. | Private Doctor | 1 | 0 |
| 10. | Supermarket | 1 | 0 |
| 11. | Filling station | 1 | 0 |
| 12. | Other (Please specify) | 1 | 0 |
|  | **6.6 How easy is it for you to buy condoms in your community?** |  |  |
| 1. | Very difficult | 0 |  |
| 2. | Quite difficult | 1 |  |
| 3. | Quite easy | 2 |  |
| 4. | Very easy | 3 |  |
| 5. | Don’t know | 4 |  |

|  | **6.7 How easy is it for you to get free condoms from clinics in your community?** | |
| --- | --- | --- |
| 1. | Very difficult | 0 |
| 2. | Quite difficult | 1 |
| 3. | Quite easy | 2 |
| 4. | Very easy | 3 |
| 5. | Don’t know | 4 |

|  | **6.8 How important is it for you to use condoms when you have sexual intercourse with a casual partner?** | |
| --- | --- | --- |
| 1. | Extremely important | 0 |
| 2. | Quite important | 1 |
| 3. | Quite unimportant | 2 |
| 4. | Extremely unimportant | 3 |
| 5. | Don’t know | 4 |
|  | **6.9 How important is it for you to use condoms when you have sexual intercourse with your regular partner?** | |
| 1. | Extremely important | 0 |
| 2. | Quite important | 1 |
| 3. | Quite unimportant | 2 |
| 4. | Extremely unimportant | 3 |
| 5. | Don’t know | 4 |
|  | **6.10 Have you talked with your partner about condoms in the past 12 months?** |  |
| 1. | No | 0 |
| 2. | Yes | 1 |
| 3. | Not applicable | 9 |

**SECTION 7: PREGNANCY EXPERIENCES**

|  | **7.1 Have you ever been pregnant?** | |
| --- | --- | --- |
| 1. | No | 0 |
| 2. | Yes | 1 |

|  | **7.2 If yes, did you intentionally want to get pregnant?** | |
| --- | --- | --- |
| 1. | No | 0 |
| 2. | Yes | 1 |
| 3. | Not applicable | 9 |

|  | **7.3 How many miscarriages have you had in total, if any?** |  |
| --- | --- | --- |
| 1. | None | 0 |
| 2. | 1 to 2 | 1 |
| 3. | 3 to 4 | 2 |
| 4. | 5 or more | 3 |

|  | **7.4 Have you ever terminated pregnancy?** | |
| --- | --- | --- |
| 1. | No | 0 |
| 2. | Yes | 1 |
| 3. | Not applicable | 9 |

**IF NEVER PREGNANT AND NEVER HAD MISCARRIAGES, PLEASE GO TO SECTION 9.**

|  | **7.5 At the time you became pregnant with your last child, how much did you want to become pregnant then?** | |  |
| --- | --- | --- | --- |
| 1. | A great deal | 1 |  |
| 2. | A little | 2 |  |
| 3. | Not much | 3 |  |
| 4. | Not at all | 4 |  |
|  | **7.6 How much longer would you like to have waited?** |  | |
| 1. |  | Months | |
| 2. |  | Years | |
| 3. | 9 | Not applicable | |

|  | **7.7 Where did you go for antenatal care the majority of times during the last pregnancy?** | |  |
| --- | --- | --- | --- |
| 1. | Public hospital | 1 |  |
| 2. | Private hospital | 2 |  |
| 3. | Public clinic | 3 |  |
| 4. | Public surgery | 4 |  |
| 5. | Private midwife’s office | 5 |  |
| 6. | Other (please specify) | 6 |  |
| 7. | Not applicable | 9 |  |
|  | **7.8 What was the outcome of the pregnancy?** |  |  |
| 1. | Full-term | 1 |  |
| 2. | Pre-term (premature) | 2 |  |
| 3. | Still-born | 3 |  |
| 4. | Voluntarily terminated pregnancy | 4 |  |
| 5. | Miscarriage | 5 |  |
|  | **7.9 Where did you give birth?** |  |  |
| 1. | Home | 1 |  |
| 2. | Government Hospital | 2 |  |
| 3. | Day hospital/clinic/community health centre | 3 |  |
| 4. | Private hospital/clinic | 4 |  |
| 5. | Other (Please specify) | 5 |  |
|  | **7.10 How old were you when you gave birth to your last child?** |  | |
| 1. |  | Years | |
| 2. | 99 | Do not know/do not remember | |

**SECTION 8: PREGNANCY AND ALCOHOL USE**

|  | **8.1 When last were you pregnant?** | | | | |  | | |  |  |  |
| --- | --- | --- | --- | --- | --- | --- | --- | --- | --- | --- | --- |
| 1. | In the past year | | | | | 1 | | |  |  |  |
| 2. | Between 1-2 years ago | | | | | 2 | | |  |  |  |
| 3. | Between 2-3 years ago | | | | | 3 | | |  |  |  |
| 4. | Between 3-4 years ago | | | | | 4 | | |  |  |  |
| 5. | Between 4-5 years ago | | | | | 5 | | |  |  |  |
| 6. | More than 5 years ago | | | | | 6 | | |  |  |  |
|  | **8.2 Did you plan to stop drinking because of the pregnancy?** | | | | |  | | |  |  |  |
| 1. | No | | | | | 0 | | |  |  |  |
| 2. | Yes | | | | | 1 | | |  |  |  |
| 3. | Not applicable/Not drinking at time of falling pregnant | | | | | 9 | | |  |  |  |
|  | **8.3 Which of the following factors made it difficult for you to stop drinking during pregnancy?** | | | | | | | | |  |  |
|  |  | Definitely true | Mostly true | | Not sure | | Mostly false | | | Definitely false | Not applicable |
| 1. | Influences from my friend(s) | 1 | 2 | | 3 | | 4 | | | 5 | 6 |
| 2. | Influences from my partner(s) | 1 | 2 | | 3 | | 4 | | | 5 | 6 |
| 3. | Influences from family member(s) | 1 | 2 | | 3 | | 4 | | | 5 | 6 |
| 4. | Stress | 1 | 2 | | 3 | | 4 | | | 5 | 6 |
| 5. | I felt addicted | 1 | 2 | | 3 | | 4 | | | 5 | 6 |
| 6. | I enjoyed drinking too much | 1 | 2 | | 3 | | 4 | | | 5 | 6 |
|  | **8.4 After you knew you were pregnant, how often did you have a drink containing alcohol?** | | | | | | |  |  |  |  |
| 1. | Never | | | 0 | | | |  |  |  |  |
| 2. | Monthly or less | | | 1 | | | |  |  |  |  |
| 3. | 2 to 4 times a month | | | 2 | | | |  |  |  |  |
| 4. | 2 to 3 times a week | | | 3 | | | |  |  |  |  |
| 5. | 4 or more times a week | | | 4 | | | |  |  |  |  |

| 1. | None | 0 |
| --- | --- | --- |
| 2. | 1 or 2 | 1 |
| 3. | 3 or 4 | 2 |
| 4. | 5 or 6 | 3 |
| 5. | 7 to 9 | 4 |
| 6. | 10 or more | 5 |
| 7. | Other, please specify. If the respondent drank homebrew please ask her to indicate the name of the homebrew, type of container, and quantity consumed. | 6 |

**8.5 After you knew you were pregnant, how many drinks containing alcohol did you have on a typical day when you were drinking?**

|  | **SECTION 9: ALCOHOL USE**   \|  \| **9.1 Have you had a drink containing alcohol in the past 3 months?** \| \| \| --- \| --- \| --- \| \| 1. \| No \| 0 \| \| 2. \| Yes \| 1 \|  \| **IF NO, PLEASE GO TO SECTION 10** \|  \| \| --- \| --- \|  \|  \| **9.2 How old were you when you first started drinking alcohol?** \| \| \| \| --- \| --- \| --- \| --- \| \| 1. \|  \| years \| \| 2. \| 1 \| Can’t remember \|  \|  \| **9.3 Do you still take a drink with alcohol sometimes?** \| \| \| --- \| --- \| --- \| \| 1. \| No \| 0 \| \| 2. \| Yes \| 1 \|  \|  \| **9.4 How often do you have a drink containing alcohol?** \| \| \| --- \| --- \| --- \| \| 1. \| 2 to 3 times a week \| 1 \| \| 2. \| 4 or more times a week \| 2 \| \| 3. \| Monthly or less \| 3 \| \| 4. \| 2 to 4 times a month \| 4 \|  \|  \| **9.5 When did you stop drinking alcohol?** \|  \| \| --- \| --- \| --- \| \| 1. \| 0-6 months ago \| 1 \| \| 2. \| 7-12 months ago \| 2 \| \| 3. \| 1-2 years ago \| 3 \| \| 4. \| 2-3 years ago \| 4 \| \| 5. \| 3 years or more \| 5 \| \| 6. \| Not applicable \| 9 \|  \|  \| **IF YOU HAVE NOT HAD AN ALCOHOLIC DRINK IN THE PAST YEAR, PLEASE GO TO SECTION 10** \| \|  \|  \|  \| \| --- \| --- \| --- \| --- \| --- \| --- \| \|  \| \| **9.6 How many drinks containing alcohol do you have on a typical day when you are drinking? (Please note that one drink is equivalent to one can or bottle of beer, cider or coolers, one glass of wine, or one tot of spirits).** \| \| \| 1. \| \| None \| 0 \| \| 2. \| \| 1 or 2 \| 1 \| \| 3. \| \| 3 or 4 \| 2 \| \| 4. \| \| 5 or 6 \| 3 \| \| 5. \| \| 7 to 9 \| 4 \| \| 6. \| \| 10 or more \| 5 \| \| 7. \| \| Other (please specify). If you drink homebrew please indicate the name of the homebrew, type of container, and quantity \| 6 \|   **SECTION 10: CULTURE**   \|  \| **10.1 According to your culture, men are entitled to have as many children as they wish to have.** \| \| \| --- \| --- \| --- \| \| 1. \| Strongly agree \| 1 \| \| 2. \| Moderately agree \| 2 \| \| 3. \| Moderately disagree \| 3 \| \| 4. \| Strongly disagree \| 4 \|  \|  \| **10.2 According to your culture, it is always, usually, sometimes or never wrong not to have children?** \| \| \| --- \| --- \| --- \| \| 1. \| Always wrong \| 1 \| \| 2. \| Usually wrong \| 2 \| \| 3. \| Sometimes wrong \| 3 \| \| 4. \| Never wrong \| 4 \|  \|  \| **10.3 According to your culture, having children is a sign that you are a worthy woman.** \| \| \| --- \| --- \| --- \| \| 1. \| Very true \| 1 \| \| 2. \| Somewhat true \| 2 \| \| 3. \| Somewhat untrue \| 3 \| \| 4. \| Very untrue \| 4 \|  \|  \| **10.4 According to your culture, for a man to have children is a sign that he is a worthy man.** \| \| \| --- \| --- \| --- \| \| 1. \| Very true \| 1 \| \| 2. \| Somewhat true \| 2 \| \| 3. \| Somewhat untrue \| 3 \| \| 4. \| Very untrue \| 4 \|   **SECTION 11: MALE PARTNERS**  **11.1 How many sexual male partners do you currently have? **  **11.2 How many sexual male partners did you have in the past year? **  **11.3 How many sexual male partners have you ever had in your lifetime? **   \|  \| **11.4 Who is your current partner?** \| \|  \| \| \| --- \| --- \| --- \| --- \| --- \| \| 1. \| No one \| \| 0 \| \| \| 2. \| Father of the child \| \| 1 \| \| \| 3. \| Someone else \| \| 2 \| \| \|  \| \| **11.5 How old is your sexual partner(s)?** \| \| --- \| \| \| \| \| \|  \| \| 1. \|  \| \| \| \| Years \| \|  \| **11.6 Is your partner employed?** \| \| \| \| \|  \| \| 1. \| No \| \| \| \| \| 0 \| \| 2. \| Yes \| \| \| \| \| 1 \| \| 3. \| Self employed \| \| \| \| \| 2 \| \|  \| **11.7 What is your sexual partner’s HIV status?** \| \| \|  \| \| \| 1. \| HIV negative \| \| \| 0 \| \| \| 2. \| HIV positive \| \| \| 1 \| \| \| 3. \| I don’t know \| \| \| 2 \| \| \|  \| **11.8 Have you ever been diagnosed with HIV positive?** \| \| \|  \| \| \| 1. \| No \| \| \| 0 \| \| \| 2. \| Yes \| \| \| 1 \| \| \|  \| **11.9 Please indicate how strongly you agree or disagree with the following statements.** \| \| \| \| \| \| \|  \|  \| \|  \|  \| Strongly agree \| \| Moderately agree \| \| \| Neither agree nor disagree \| Moderately disagree \| Strongly disagree \| \| 1. \| You are satisfied with your relationship with this person \| 1 \| \| 2 \| \| \| 3 \| 4 \| 5 \| \| 2. \| Sometimes there is serious disagreements between you and him \| 1 \| \| 2 \| \| \| 3 \| 4 \| 5 \| \| 3. \| Sometimes there is hitting or slapping between you and him \| 1 \| \| 2 \| \| \| 3 \| 4 \| 5 \| \| 4. \| You have a lot of control in your relationship with him \| 1 \| \| 2 \| \| \| 3 \| 4 \| 5 \| \| 5. \| There is a lot of trust between you and him \| 1 \| \| 2 \| \| \| 3 \| 4 \| 5 \| \| 6. \| Your partner has a control of whether or not to have sex \| 1 \| \| 2 \| \| \| 3 \| 4 \| 5 \| \| 7. \| Your partner has control of whether or not a condom is used \| 1 \| \| 2 \| \| \| 3 \| 4 \| 5 \| \| 8. \| Your partner has control of whether or not a contraceptive method is used for birth control \| 1 \| \| 2 \| \| \| 3 \| 4 \| 5 \|   **11.10 Now I would like to ask about his drinking of alcoholic beverages.**   \|  \|  \| Never \| Less than monthly \| Monthly \| Weekly \| Daily or almost daily \| \| --- \| --- \| --- \| --- \| --- \| --- \| --- \| \| 1. \| How often does he have a drink containing alcohol? \| 0 \| 1 \| 2 \| 3 \| 4 \| \| 2. \| How often do you drink with him? \| 0 \| 1 \| 2 \| 3 \| 4 \| \| 3. \| How often does he have six or more drinks on one occasion? \| 0 \| 1 \| 2 \| 3 \| 4 \|   **11.11 Now I would like to ask about the effect of his drinking of alcoholic beverages**   \|  \|  \| No \| Yes \| Don't know \| \| --- \| --- \| --- \| --- \| --- \| \| 1. \| Has he or someone else ever injured as a result of his drinking? \| 0 \| 1 \| 2 \| \| 2. \| Did a relative, friend, or a doctor or other health worker ever express concern about his drinking or suggest that he cut down? \| 0 \| 1 \| 2 \|  \|  \| **11.12 How many drinks containing alcohol does he have on a typical day when he is drinking?** \| \| \| --- \| --- \| --- \| \| 1. \| None \| 0 \| \| 2. \| 1 or 2 \| 1 \| \| 3. \| 3 or 4 \| 2 \| \| 4. \| 5 or 6 \| 3 \| \| 5. \| 7 to 9 \| 4 \| \| 6. \| 10 or more \| 5 \|  \|  \| **11.13 Do you feel obliged to drink alcohol when your partner is drinking?** \| \| \| --- \| --- \| --- \| \| 1. \| No \| 0 \| \| 2. \| Yes \| 1 \|  \|  \| **11.14 Have you ever experienced forced sex with your partner?** \| \| \| --- \| --- \| --- \| \| 1. \| No \| 0 \| \| 2. \| Yes \| 1 \|   **SECTION 12: HEALTHCARE FACILITY**   \|  \| **12.1 How often do you go to clinic?** \|  \| \| --- \| --- \| --- \| \| 1. \| More than 1 time(s) a month \| 1 \| \| 2. \| 1 time each month \| 2 \| \| 3. \| Once in 2 months \| 3 \| \| 4. \| Once in 3 - 5 months \| 4 \| \| 5. \| Once in more than 5 months, but less than a year \| 5 \| \| 6. \| Once a year \| 6 \|  \|  \| **12.2 Please indicate how strongly you agree or disagree with the following statements.** \| Strongly agree \| Moderately agree \| Neither agree nor disagree \| Moderately disagree \| Strongly disagree \| \| --- \| --- \| --- \| --- \| --- \| --- \| --- \| \| 1. \| Is it easy to access contraceptives at my nearest clinic \| 1 \| 2 \| 3 \| 4 \| 5 \| \| 2. \| Nurses at the clinic know their work \| 1 \| 2 \| 3 \| 4 \| 5 \| \| 3. \| Nurses at the clinic provide counselling and education about contraception each time I visit the clinic \| 1 \| 2 \| 3 \| 4 \| 5 \| \| 4. \| Nurses at the clinic are friendly and open despite my age and HIV status \| 1 \| 2 \| 3 \| 4 \| 5 \| \| 5. \| I do not feel judged at all by nurses at the clinic \| 1 \| 2 \| 3 \| 4 \| 5 \| \| 6. \| The waiting hours are long at the clinic \| 1 \| 2 \| 3 \| 4 \| 5 \| \| 7. \| The waiting hours are unbearable at the clinic \| 1 \| 2 \| 3 \| 4 \| 5 \| \| 8. \| My contraceptive method of choice is always available at the clinic \| 1 \| 2 \| 3 \| 4 \| 5 \|   **THE END.**  **THANK YOU FOR YOUR PARTICIPATION.** |
| --- | --- | --- | --- | --- | --- | --- | --- | --- | --- | --- | --- | --- | --- | --- | --- | --- | --- | --- | --- | --- | --- | --- | --- | --- | --- | --- | --- | --- | --- | --- | --- | --- | --- | --- | --- | --- | --- | --- | --- | --- | --- | --- | --- | --- | --- | --- | --- | --- | --- | --- | --- | --- | --- | --- | --- | --- | --- | --- | --- | --- | --- | --- | --- | --- | --- | --- | --- | --- | --- | --- | --- | --- | --- | --- | --- | --- | --- | --- | --- | --- | --- | --- | --- | --- | --- | --- | --- | --- | --- | --- | --- | --- | --- | --- | --- | --- | --- | --- | --- | --- | --- | --- | --- | --- | --- | --- | --- | --- | --- | --- | --- | --- | --- | --- | --- | --- | --- | --- | --- | --- | --- | --- | --- | --- | --- | --- | --- | --- | --- | --- | --- | --- | --- | --- | --- | --- | --- | --- | --- | --- | --- | --- | --- | --- | --- | --- | --- | --- | --- | --- | --- | --- | --- | --- | --- | --- | --- | --- | --- | --- | --- | --- | --- | --- | --- | --- | --- | --- | --- | --- | --- | --- | --- | --- | --- | --- | --- | --- | --- | --- | --- | --- | --- | --- | --- | --- | --- | --- | --- | --- | --- | --- | --- | --- | --- | --- | --- | --- | --- | --- | --- | --- | --- | --- | --- | --- | --- | --- | --- | --- | --- | --- | --- | --- | --- | --- | --- | --- | --- | --- | --- | --- | --- | --- | --- | --- | --- | --- | --- | --- | --- | --- | --- | --- | --- | --- | --- | --- | --- | --- | --- | --- | --- | --- | --- | --- | --- | --- | --- | --- | --- | --- | --- | --- | --- | --- | --- | --- | --- | --- | --- | --- | --- | --- | --- | --- | --- | --- | --- | --- | --- | --- | --- | --- | --- | --- | --- | --- | --- | --- | --- | --- | --- | --- | --- | --- | --- | --- | --- | --- | --- | --- | --- | --- | --- | --- | --- | --- | --- | --- | --- | --- | --- | --- | --- | --- | --- | --- | --- | --- | --- | --- | --- | --- | --- | --- | --- | --- | --- | --- | --- | --- | --- | --- | --- | --- | --- | --- | --- | --- | --- | --- | --- | --- | --- | --- | --- | --- | --- | --- | --- | --- | --- | --- | --- | --- | --- | --- | --- | --- | --- | --- | --- | --- | --- | --- | --- | --- | --- | --- | --- | --- | --- | --- | --- | --- | --- | --- | --- | --- | --- | --- | --- | --- | --- | --- | --- | --- | --- | --- | --- | --- | --- | --- | --- | --- | --- | --- | --- | --- | --- | --- | --- | --- | --- | --- | --- | --- | --- | --- | --- | --- | --- | --- | --- | --- | --- | --- | --- | --- | --- | --- | --- | --- | --- | --- | --- | --- | --- | --- | --- | --- | --- | --- | --- | --- | --- | --- | --- | --- | --- | --- | --- | --- | --- | --- | --- | --- | --- | --- | --- | --- | --- | --- | --- | --- | --- | --- | --- | --- | --- | --- | --- | --- | --- | --- | --- | --- | --- | --- | --- | --- | --- | --- | --- | --- | --- | --- | --- | --- | --- | --- | --- | --- | --- | --- | --- | --- | --- | --- | --- | --- | --- | --- | --- | --- | --- | --- | --- | --- | --- | --- | --- | --- | --- | --- | --- | --- | --- | --- | --- | --- | --- | --- | --- | --- | --- | --- | --- | --- | --- | --- | --- | --- | --- | --- | --- | --- | --- | --- | --- | --- | --- | --- | --- | --- | --- | --- | --- | --- | --- | --- | --- | --- | --- |
